# Temporary caval occlusion technique during right divisional hepatectomy for massive hepatocellular carcinoma with caudal vena cava compression/involvement in dogs

**DOI:** 10.1111/vsu.70133

**Published:** 2026-07-08

**Authors:** Kumiko Ishigaki, Kazushi Asano, Kei Tamura, Tatsuya Heishima, Takeshi Hayakawa, Teppei Fujimoto, Kenji Teshima

**Affiliations:** ^1^ Department of Veterinary Medicine, College of Bioresource Sciences Nihon University Fujisawa Japan; ^2^ Present address: Animal Emergency Medical Center Tokyo University of Agriculture and Technology Tokyo Japan

## Abstract

**Objective:**

To describe the perioperative and long‐term outcomes of dogs with massive hepatocellular carcinoma (HCC) in the right hepatic division causing compression or involvement of the caudal vena cava (CVC) treated with right divisional hepatectomy using temporary caval occlusion (TCO).

**Study design:**

Retrospective case series.

**Animals:**

Nineteen client‐owned dogs.

**Methods:**

Medical records were reviewed. Data collected included signalment, preoperative imaging findings, operative details, adjunct procedures, transfusion requirements, histopathological diagnosis and surgical margin status, perioperative complications, and survival to discharge. Long‐term outcome information was obtained from recheck examinations when available and from telephone interviews with referring veterinarians and/or owners.

**Results:**

Right divisional hepatectomy with TCO was completed in all dogs; a Pringle maneuver was used in two of 19 dogs. Median TCO duration was 17.6 min (range, 7.0–25.7) and median operative time was 82 min (range, 69–127). Primary suture repair of the CVC was required in six of 19 dogs. R0 resection was achieved in 14 of 19 dogs. One dog died on postoperative day 2; 18/19 dogs were discharged. Of these dogs, seven were alive without reported recurrence or intrahepatic metastasis at the last follow up, 95–1355 days after surgery, whereas 11 had died at a median of postoperative day 729 (range, 204–1154). Postoperative intrahepatic metastasis was suspected in two dogs.

**Conclusion:**

Temporary caval occlusion‐assisted right divisional hepatectomy provided venous control in selected dogs with massive right‐sided HCC and compression or involvement of the CVC, with no intraoperative mortality, one in‐hospital death, and postoperative complications in six of the 18 discharged dogs.

**Clinical significance:**

Temporary caval occlusion facilitates controlled dissection of the right hepatic veins and repair of small caval defects when present.

**Abbreviations**
3D: three‐dimensional
CBC: complete blood count
CRI: constant‐rate infusion
CT: computed tomography
CVC: caudal vena cava
HCC: hepatocellular carcinoma
Postop: postoperative
R0: microscopically negative margin
R1: microscopically positive margin
R2: macroscopically residual tumor
TCO: temporary caval occlusion

## 1 INTRODUCTION

Primary hepatic tumors account for approximately 0.6% to 1.5% of all cancers in dogs.^1^, ^2^, ^3^, ^4^ Primary hepatocellular tumors include hepatocellular carcinoma (HCC), hepatocellular adenoma, and hepatoblastoma. Among these, HCC is the most common, representing up to 77% of primary hepatic tumors in dogs.^2^ Hepatocellular carcinoma most commonly presents as the massive type, characterized by a large solitary mass arising from a single hepatic lobe.^1^, ^2^, ^3^, ^4^ For dogs with massive HCC, surgical resection is considered the treatment of choice.^2^, ^3^, ^4^, ^5^ Reported rates of metastasis for massive HCC range from 0% to 37% and rates of local recurrence range from 0% to 13%, indicating a generally favorable postoperative prognosis.^5^

Several studies have identified an association between tumor location and outcomes in dogs with HCC.^5^, ^6^ Long‐term survival after resection of left divisional HCC is generally longer than that for right divisional disease.^5^ This disparity is likely attributable in part to the greater technical difficulty of surgery in the right division, with intraoperative mortality reported as high as 40% in one series.^5^ Another study also reported that intraoperative complications, including severe hemorrhage, occurred significantly more often during right divisional hepatectomy than during left divisional hepatectomy (33.3% vs. 5.6%).^6^ More recently, a multi‐institutional retrospective study of 70 dogs undergoing right divisional hepatic lobectomy reported no intraoperative deaths; however, perioperative and 30 day mortality risks were 2.9% and 12.1%, respectively.^7^ These contemporary data suggest that, although intraoperative mortality may be lower than historically reported, right divisional resections remain associated with substantial hemorrhagic and hemodynamic risk, particularly when lesions are closely associated with, compress, or involve the caudal vena cava (CVC). These vulnerabilities are attributed to the close anatomic relationship of the main and accessory right hepatic veins to the CVC within the right divisional parenchyma,^8^ which predisposes to venous back bleeding that is not controlled by inflow occlusion alone (e.g., the Pringle maneuver). Accordingly, an increased risk of intraoperative complications should be anticipated when surgical resection of right divisional HCC is planned. In particular, when a large right divisional HCC compresses or involves the CVC, the risk of venous back bleeding from the CVC increases. Operative strategies that proactively reduce hemorrhage during dissection of the right hepatic veins should therefore be employed in such cases.

Temporary caval occlusion (TCO) is a venous control maneuver in which cranial and caudal tourniquets are used to transiently occlude the CVC, minimizing back bleeding during hepatic vein dissection. This approach is conceptually related to established methods of vascular control and is supported by published data on the physiologic tolerance of short periods of CVC occlusion in dogs.^9^ The objectives of this study were to describe the authors’ institutional management and the perioperative and long‐term outcomes of dogs with massive right divisional HCC and CVC compression or involvement that underwent right divisional hepatectomy using TCO.

## 2 MATERIALS AND METHODS

### 2.1 Case selection and preoperative planning

#### 2.1.1 Study population

The study population comprised client‐owned dogs, treated as part of standard clinical care, that underwent right divisional hepatectomy with TCO at the authors’ hospital between May 2021 and December 2025. Dogs with right divisional hepatic masses that did not undergo TCO‐assisted right divisional hepatectomy were not included in the outcome analysis. Dogs were eligible when computed tomography (CT) demonstrated a large mass in the right hepatic division compressing or involving the CVC, with no evidence of extrahepatic metastasis. Dogs with an additional resectable lesion in another hepatic lobe were eligible; when indicated, concurrent lobectomy of that lobe was performed. Cases with diffuse or unresectable multifocal hepatic disease were excluded. Medical records were reviewed for signalment, physical examination findings, complete blood count (CBC), serum chemistry, thoracic radiography, abdominal ultrasonography, and CT findings.

Written owner consent for surgery and for the use of anonymized medical records and imaging for research and publication was obtained for all dogs.

#### 2.1.2 Computed tomography protocol and measurement of CVC compression

Multidetector CT was performed with and without intravenous iodinated contrast using a power injector. Images were acquired in arterial, venous, and delayed phases under end‐expiratory breath hold, and reconstructed at 1 mm slice thickness. The degree of CVC compression was assessed on the venous phase and calculated as: CVC compression (%) = [1 − (minimal short‐axis diameter of the CVC at the point of maximal tumor‐induced compression/reference short‐axis diameter of the intrathoracic CVC measured immediately cranial to the diaphragmatic caval foramen in an uninvolved segment)] × 100. Figures 1 and 2 show representative preoperative CT images demonstrating the relationship between the right divisional hepatic mass, CVC compression, and right hepatic venous anatomy.

**FIGURE 1:**
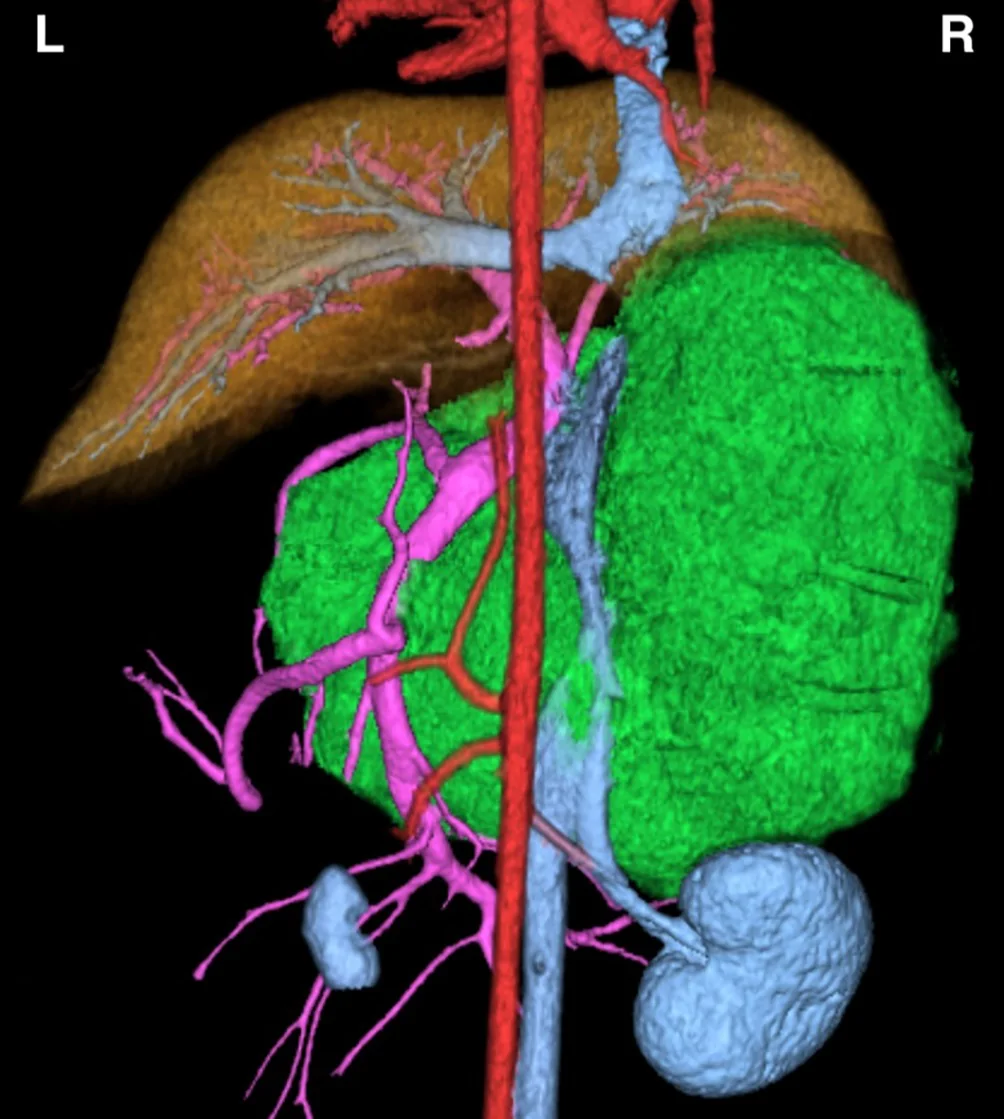
Preoperative computed tomography (CT) three‐dimensional (3D) reconstruction in a representative case. Dorsal view of the 3D reconstruction showing a mass compressing and partially involving the caudal vena cava (CVC). Color key: red, heart and aorta; brown, normal hepatic parenchyma; green, hepatocellular carcinoma (HCC); blue, CVC, kidneys, and renal veins; purple, portal veins.

**FIGURE 2:**
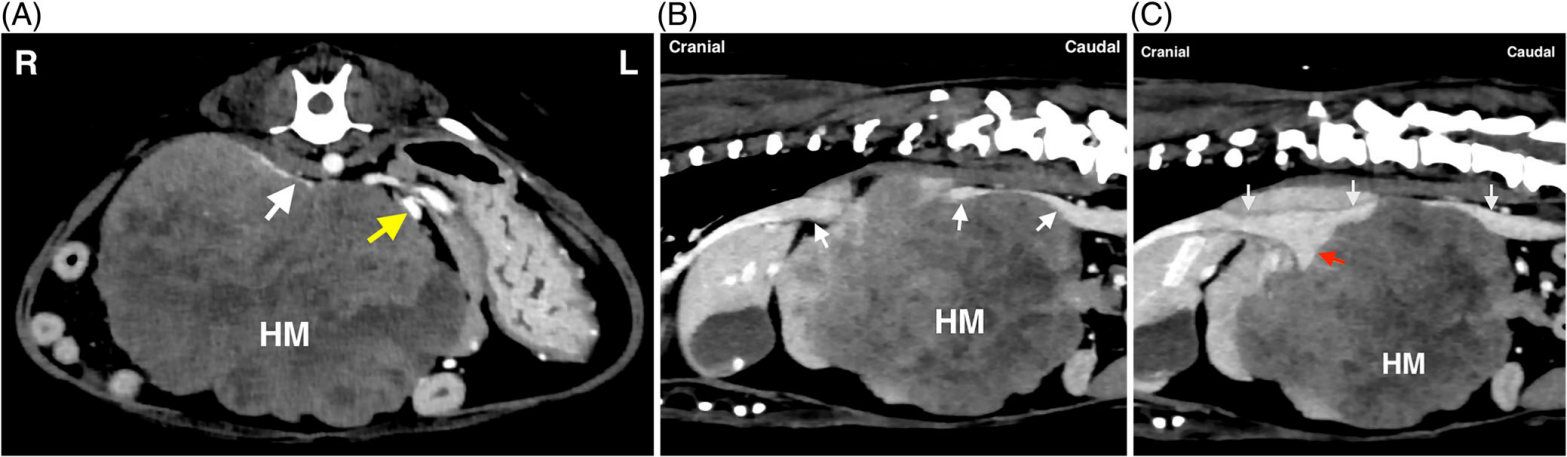
Preoperative venous‐phase computed tomography (CT) in the same dog as in Figure 1. (A) Axial image at the level of maximal tumor‐induced deformation showing severe compression of the caudal vena cava (CVC). (B) Sagittal image demonstrating the relationship between the right divisional mass and the CVC. In this dog, CVC compression was calculated as 85.4%. (C) Sagittal image demonstrating the relationship between the right divisional mass and the right hepatic vein. Images were obtained during an end‐expiratory breath hold. HM indicates right divisional hepatic mass; white arrows indicate the CVC; the yellow arrow indicates the main portal vein; the red arrow indicates the right hepatic vein.

### 2.2 Anesthesia and perioperative management

The anesthesia protocol included subcutaneous prednisolone 1.0 mg/kg, maropitant 1.0 mg/kg, and fentanyl 5.0 μg/kg. General anesthesia was induced with intravenous propofol and maintained with sevoflurane in oxygen with mechanical ventilation. Vasoactive support during and after surgery included dopamine 1.25–10 μg/kg/min, dobutamine 1.25–10 μg/kg/min, nafamostat mesylate (Fusan; Nichi‐Iko Co., Ltd., Toyama, Japan) 0.1–0.2 mg/kg/h, and carperitide (HANP; Daiichi Sankyo Co., Ltd., Tokyo, Japan) 0.05–0.2 μg/kg/min, administered as clinically indicated. Analgesia consisted of intraoperative and postoperative constant‐rate infusions (CRIs) of fentanyl 2.5–20 μg/kg/h and lidocaine 25–75 μg/kg/min. For additional postoperative analgesia, ultrasound‐guided paravertebral blocks were performed bilaterally at the 9th, 11th, and 13th thoracic levels with bupivacaine, 0.25 mg/kg per site. Substantial hemorrhage was anticipated during the dissection of the mass from the major vessels because of the tumor's size, compression, or involvement of the CVC, and the need for extensive hepatic venous dissection under TCO. A donor dog was therefore kept on standby, and transfusion was administered only when clinically indicated based on intraoperative hemorrhage and hemodynamic status, at the discretion of the anesthesia and surgical teams.

### 2.3 Surgical technique for right divisional hepatectomy

#### 2.3.1 Patient positioning and surgical approach

Each dog was positioned in dorsal recumbency under general anesthesia. A central venous catheter was placed percutaneously in the right jugular vein. A Mercedes incision (cranial midline celiotomy with bilateral paracostal extensions) was performed (Figure 3A). When exposure was inadequate, a caudal median sternotomy and a midline diaphragmatic incision were added. Adhesions between the hepatic mass and surrounding tissues were released by adhesiolysis using a monopolar electrocautery and a vessel sealing system (ForceTriad; Medtronic plc, Dublin, Ireland). The right triangular ligaments were then fully divided.

**FIGURE 3:**
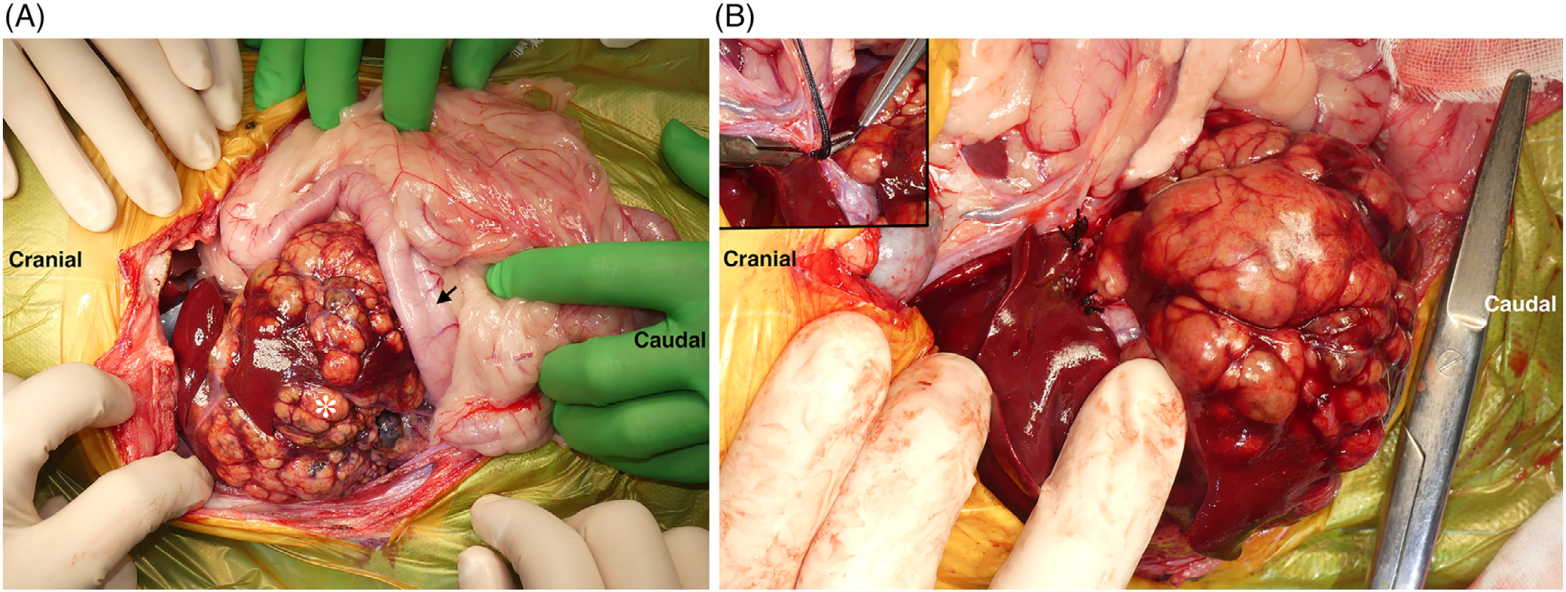
Hepatic inflow transection during right divisional hepatectomy in the same dog as in Figures 1 and 2. (A) Mercedes incision and exposure of the cranial abdomen. The asterisk indicates the right divisional hepatic mass; the black arrow indicates the duodenum. (B) En masse ligation and transection of the right divisional Glissonian pedicle. The inset (upper left) shows en masse ligation with 0 braided nylon using right‐angle forceps.

#### 2.3.2 Control of hepatic inflow

To control inflow to the right hepatic division, the Glissonian sheath to the right division, containing the right hepatic arterial branches, the right branch of the portal vein, and the right hepatic duct, was isolated, encircled, and ligated en masse with two to four sutures of 0 braided coated nylon (Surgilon; Medtronic, Minneapolis, Minnesota) and/or 3–0 polydioxanone (PDS II; Ethicon, Johnson & Johnson, New Brunswick, New Jersey) using right‐angle forceps (Figure 3B); the pedicle was then divided between ligatures. When isolation of the right divisional Glissonian pedicle was challenging due to tumor adhesion or involvement, dissection and ligation were performed using an ultrasonic surgical aspirator (SonoSurg; Olympus, Tokyo, Japan) during a Pringle maneuver to achieve temporary inflow occlusion as clinically indicated.

#### 2.3.3 Temporary caval occlusion

After inflow control, the hepatorenal ligament was incised to its attachment on the CVC. The retroperitoneum along the right diaphragmatic crus and adjacent dorsal musculature was transected, exposing the dorsal aspect of the CVC (Figure 4A). An umbilical tape was passed around the CVC between the right hepatic division and the right adrenal gland to create a caudal tourniquet. When the right adrenal gland was involved by the tumor or enlarged, the caudal tourniquet was placed caudal to the gland (Figure 4B). Right‐angle forceps were then gently advanced bluntly between the right medial lobar Glisson's sheath and the CVC (Figure 4C). The tips were passed dorsally around the CVC, cranial to the right division near the diaphragm (Figure 4D). The cranial umbilical tape was grasped and brought through to complete a cranial tourniquet. The CVC was occluded by tightening the caudal tourniquet first, followed by the cranial tourniquet (Figure 4E). Temporary caval occlusion duration was defined as the interval from tightening of the caudal tourniquet to release of the cranial tourniquet.

**FIGURE 4:**
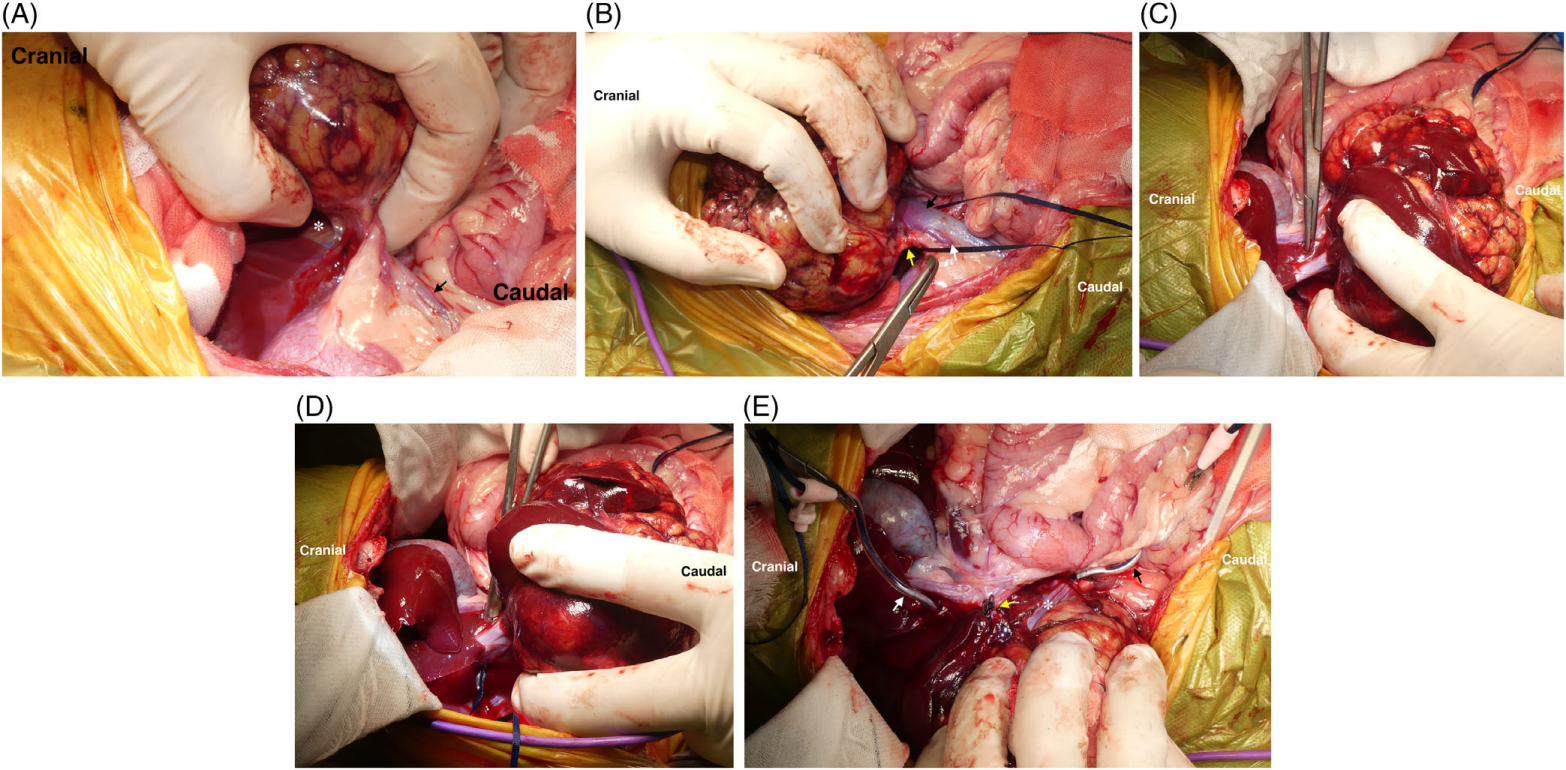
Temporary caval occlusion (TCO) during right divisional hepatectomy in the same dog as in Figure 3. (A) Opening the dorsal aspect of the right hepatic division by incising the hepatorenal ligament and the retroperitoneum along the right diaphragmatic crus. The asterisk indicates the dorsal aspect of the mass. A black arrow indicates the caudal vena cava (CVC). (B) Placement of a caudal tourniquet around the CVC caudal to the right hepatic division and the right adrenal gland. In this case, the right adrenal gland was firmly adherent to the mass and en bloc removal with the right hepatic division was planned. The black arrow indicates the CVC; the white arrow indicates the right renal vein; the yellow arrow indicates the right adrenal gland. (C) Insertion of right‐angle forceps between the right medial lobar Glissonian pedicle and the CVC. (D) Retrieval of a cranial umbilical tape using the right‐angle forceps around the CVC near the diaphragm to complete tourniquet placement. (E) Establishment of TCO by tightening the caudal tourniquet followed by the cranial tourniquet. The asterisk indicates the CVC; the white arrow indicates the cranial tourniquet; the black arrow indicates the caudal tourniquet; the yellow arrow indicates the transected right divisional Glissonian pedicle.

#### 2.3.4 En bloc resection and control of hepatic outflow

With TCO in place, dissection of the right hepatic division from the CVC was performed using an ultrasonic surgical aspirator and a vessel sealing system. At the junctions of the accessory right hepatic vein and the right hepatic vein with the CVC, each vein was individually isolated, ligated with two to four sutures of 3–0 polydioxanone, and divided using Kaye–Freeman or microsurgical scissors (Figure 5A). For larger veins, 0 braided nylon was used. After all right hepatic venous tributaries branching from the CVC were controlled, the right hepatic division was separated from surrounding tissues, so en bloc resection was achieved (Figure 5B). The cranial and caudal tourniquets were then released to re‐establish CVC flow (Figure 5C). When bleeding from the CVC was observed, both tourniquets were reapplied to re‐establish TCO, and the bleeding point was sutured with 5–0 polypropylene (PROLENE HEMOSEAL; Ethicon, Johnson & Johnson) until hemostasis was achieved; tourniquets were then released and inspection was repeated until no further bleeding was evident. Both tourniquets were removed, and the site of the cranial encirclement was inspected for bleeding.

**FIGURE 5:**
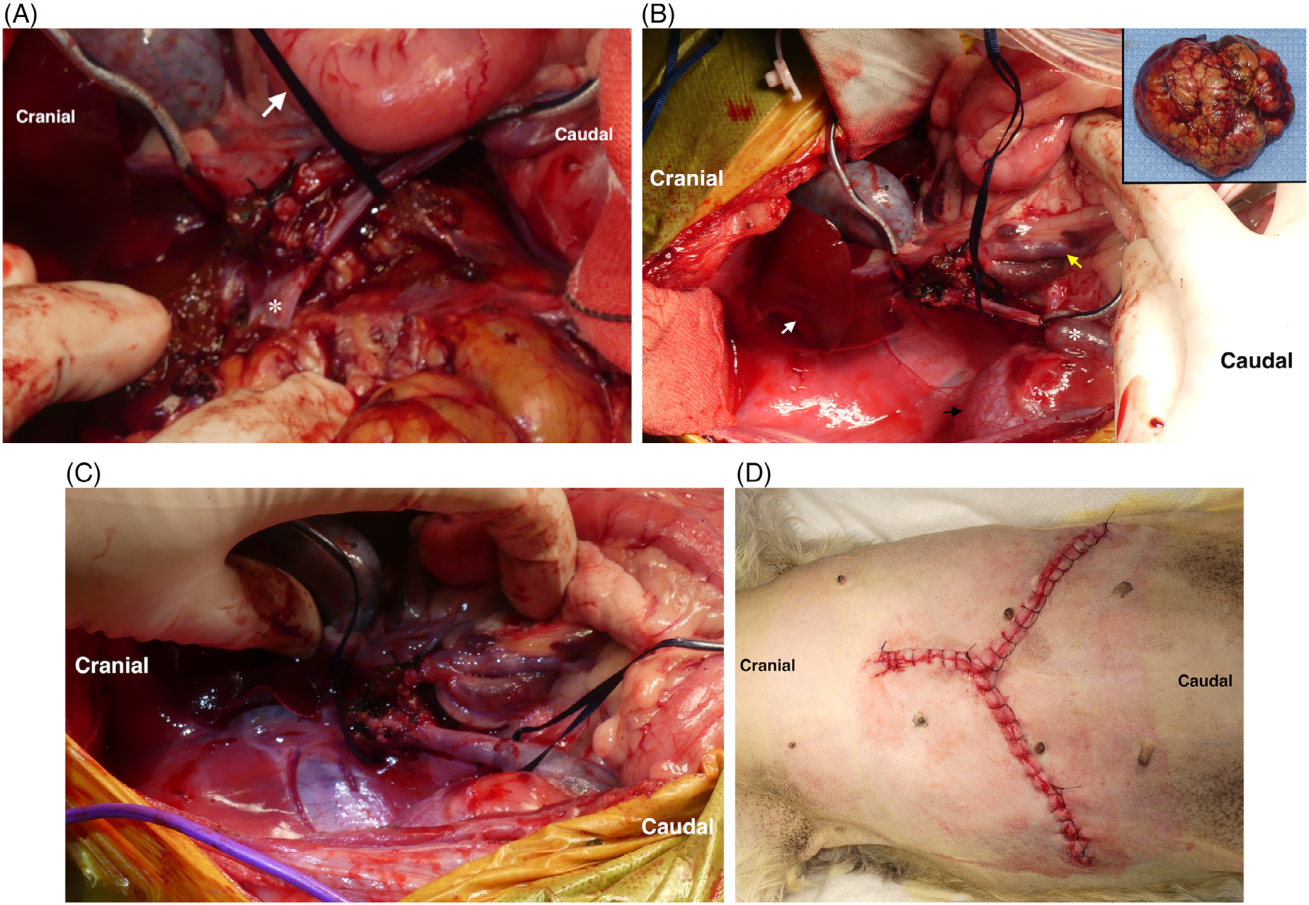
Hepatic outflow transection and en bloc resection in the same dog as in Figures 3 and 4. (A) Dissection and ligation of the main right hepatic vein at its junction with the caudal vena cava (CVC). The umbilical tape (white arrow) was used to retract the CVC gently. An ultrasonic surgical aspirator and a vessel sealing system were used as required for parenchymal dissection. The asterisk indicates the CVC. (B) Gross appearance of the CVC after en bloc resection of the right hepatic division including the mass and the right adrenal gland while both tourniquets remain tightened. The inset (upper right) shows the resected mass (mass weight 240 g; tumor‐to‐bodyweight ratio 4.6% in this case). The asterisk indicates the CVC; the white arrow indicates the right medial hepatic lobe; the black arrow indicates the right kidney; the yellow arrow indicates the portal vein. (C) Inspection for bleeding from the CVC after release of both tourniquets and re‐establishment of CVC flow; no bleeding was observed in this case. (D) Gross appearance after closure of the Mercedes incision.

#### 2.3.5 Hemostasis and closure

The abdominal cavity was thoroughly lavaged with warm sterile saline, and hemostasis was confirmed. A topical hemostatic agent (Integran; Nippon Zoki Pharmaceutical Co., Ltd., Osaka, Japan) was applied to the transection surface and the hilar dissection area. After a caudal median sternotomy and diaphragmatic incision were performed, a thoracostomy tube was placed through the abdominal wall and diaphragm into the thoracic cavity before routine thoracic and abdominal closure. The Mercedes incision was then closed routinely (Figure 5D).

### 2.4 Histopathologic examination

Specimens from resected hepatic masses were fixed in 10% neutral buffered formalin for 48 h and embedded in paraffin. Sections 4 μm thick were deparaffinized, rehydrated through graded ethanol, and stained with hematoxylin and eosin. The histologic margin status was classified as R0 (microscopically negative), R1 (microscopically positive), or R2 (macroscopically residual tumor).^10^, ^11^

### 2.5 Postoperative evaluation and outcomes

The dogs were monitored during hospitalization with physical examination, CBC, serum chemistry, and abdominal ultrasonography as indicated. Primary outcomes were completion of the planned right divisional hepatectomy using TCO, intraoperative mortality, and survival to discharge. Secondary outcomes comprised perioperative complications, TCO duration, total operative time, histologic margin status, and total perioperative blood transfusion volume. Long‐term outcomes were assessed for dogs that survived to discharge. They included survival time and evidence of recurrence or metastasis. After discharge, most dogs returned to their referring veterinarians. Dogs with suspected recurrence or intrahepatic metastasis were re‐evaluated at the authors’ hospital and underwent contrast‐enhanced CT.

### 2.6 Data collection

Variables recorded included signalment, clinical signs, laboratory results, CT findings, intraoperative findings, complications, hospital course, histopathology, and outcome. Computed tomography variables included tumor size and CVC compression as defined above. Intraoperative variables included use and duration of the Pringle maneuver, TCO duration, total operative time (skin incision to skin closure), additional procedures, intraoperative complications including CVC repair, and blood transfusion volume. Resected mass size, histopathologic diagnosis, and surgical margin status were also recorded. Long‐term outcome was assessed from the medical record when available and by telephone interviews with referring veterinarians and/or owners. Interviews focused on survival status, suspected or confirmed recurrence or metastasis, clinical course after discharge (including signs compatible with hepatic insufficiency), and cause of death when applicable.

### 2.7 Statistical analysis

Statistical analyses were performed with commercially available software (GraphPad Prism, version 10.5; GraphPad Software, San Diego, California). Continuous variables were summarized as median (range), and categorical variables as counts and percentages. The association between the degree of CVC compression and TCO duration was assessed using Spearman's rank correlation coefficient. Differences in age, body weight, TCO duration, and operative time between dogs that developed postoperative azotemia and those that did not were evaluated with the Mann–Whitney U test. Postoperative survival time was calculated from the date of surgery to the date of death or last follow up and summarized descriptively. All tests were two‐tailed, and statistical significance was set at *p* < .05. These statistical analyses were exploratory and hypothesis‐generating; no adjustment for multiple comparisons was performed because of the small sample size and retrospective case‐series design.

## 3 RESULTS

### 3.1 Signalment

During the study period, 46 dogs with right divisional hepatic masses underwent surgical resection at the hospital. Twenty‐seven of these underwent resection without TCO, and the remaining 19 underwent right divisional hepatectomy with TCO and met the inclusion criteria. In these 19 dogs, median age at presentation was 12.1 years (range, 4.9–15.6) and median body weight was 6.4 kg (range, 2.3–13.2). There were nine males (eight neutered, one intact) and 10 spayed females. Breeds were shih tzu (*n* = 7), toy poodle (*n* = 3), and one each of Jack Russell terrier, miniature dachshund, mixed breed, Pekingese, Polish lowland sheepdog, Scottish terrier, Shiba Inu, standard poodle, and Yorkshire terrier.

### 3.2 Clinical signs

Preoperative clinical signs were observed in 10 of 19 dogs and included anorexia (*n* = 3), abdominal distention (*n* = 2), vomiting (*n* = 2), concurrent polyuria and polydipsia (*n* = 2), ataxia (*n* = 1), ascites (*n* = 1), diarrhea (*n* = 1), and purpura (*n* = 1). The remaining 9 of 19 dogs were asymptomatic.

### 3.3 Preoperative blood tests

Preoperative CBC, serum chemistry, and coagulation profiles are summarized in Supporting Information, Table S1. Anemia was detected in seven of 19 (36.8%) dogs; three had severe anemia (packed cell volume 27% to 28%) with a history of intra‐abdominal hemorrhage at the referring hospital. Thrombocytosis (> 500 × 10^3^/μL) was present in 10 of 19 (52.6%); the remaining dogs had platelet counts within reference intervals.

Serum alanine aminotransferase and alkaline phosphatase were above reference intervals in 18 of 19 dogs (94.7%) and 18 of 19 dogs (94.7%), respectively. Aspartate transaminase was increased in 12 of 19 dogs (63.2%) and gamma‐glutamyl transferase in seven of 19 (36.8%). One dog had hypoglycemia (70 mg/dL). No dog had hyperbilirubinemia. Blood urea nitrogen was below reference intervals in two dogs and above in five dogs. C‐reactive protein was increased in 10 of 19 dogs (52.6%).

On coagulation testing, activated partial thromboplastin time was prolonged in three dogs, and prothrombin time was prolonged in one dog. Fibrinogen and d‐dimer were increased in seven of 19 (36.8%) and 11 of 19 dogs (57.9%), respectively.

### 3.4 Preoperative diagnostic imaging

In all 19 dogs, survey radiographs showed increased soft‐tissue opacity in the cranial abdomen, and abdominal ultrasonography identified a large hepatic mass. Thoracic radiographs showed no evidence of pulmonary metastasis in any dog.

Preoperative CT findings are summarized in Table 1. Computed tomography identified a very large mass in the right hepatic division in all dogs. All masses compressed or involved the CVC; median CVC compression was 64.7% (range, 8.9% to 90.5%) (Table 1). The main and accessory right hepatic veins were displaced and partially embedded within the right divisional parenchyma, often coursing within the mass. No dog had evidence of regional lymph node or pulmonary metastasis. Median mass volume on CT was 610.9 cm^3^ (range, 161.1–1511.9) (Table 1). One dog had an additional lesion in the left lateral lobe, which was planned for and resected concurrently.

**TABLE 1:** Computed tomography (CT) findings, intraoperative variables, and postoperative outcomes for all dogs.

Case	CT findings		Intraoperative variables						Postoperative outcomes		
	Tumor volume (cm^3^)	CVC compression (%)	Operative time (min)	Pringle time (min)	TCO time (min)	Blood transfusion (mL)	CVC repair	Mass‐to‐body weight (%)	Margin status	Recurrence/intrahepatic metastasis	Postop survival (days)
1	552.8	13.6	75	‐	14.6	0	No	4.4	R0	No	1022
2	500.8	61.3	126	‐	7.0	160	No	6.3	R0	Yes	562
3	1427.3	80.9	96	4.9	20.6	200	Yes	9.9	R0	No	875
4	192.5	51.4	82	‐	12.4	100	No	1.6	R0	No	1355+
5	161.1	8.9	81	‐	12.5	100	No	1.6	R0	No	732
6	522.1	72.5	110	4.1	10.0	100	No	7.6	R0	No	1154
7	1511.9	80.2	98	‐	25.7	200	No	5.0	R1	Yes	1151
8	610.9	90.5	72	‐	17.6	150	Yes	7.8	R0	No	632
9	497.3	43.5	80	‐	14.1	200	Yes	6.2	R0	No	457
10	164.4	85.9	90	‐	19.0	100	No	3.4	R1	No	729
11	637.1	76.5	79	‐	23.8	100	No	4.5	R0	No	397
12	631.9	57.0	86	‐	25.6	200	No	3.1	R0	No	697+
13	467.7	63.8	79	‐	16.3	100	Yes	3.9	R0	No	690+
14	1256.5	50.0	76	‐	17.9	200	Yes	5.5	R0	No	204
15	245.0	64.7	102	‐	21.1	100	No	3.5	R0	No	2
16	898.8	68.9	127	‐	13.8	400	No	3.9	R1	No	445+
17	628.4	34.8	69	‐	17.8	200	No	4.0	R0	No	231+
18	706.1	85.4	88	‐	25.3	30	No	4.6	R1	No	175+
19	768.4	83.6	77	‐	13.6	100	Yes	7.4	R1	No	95+

*Note*: Blood transfusion indicates total perioperative transfusion volume (mL) as recorded. Margin status is classified as R0 (microscopically negative) and R1 (microscopically positive). A dash indicates “not performed.” A “+” after postoperative survival indicates the dog was alive at the last follow up (censored).

Abbreviations: CT, computed tomography; CVC, caudal vena cava; Postop, postoperative; TCO, temporary caval occlusion.

### 3.5 Intraoperative findings

Right divisional hepatectomy was completed in all 19 dogs. Table 1 summarizes the intraoperative variables. The Pringle maneuver was used in two dogs for 4.1 and 4.9 min; the remaining 17 dogs did not require inflow occlusion to ligate the right divisional Glissonian pedicle. Temporary caval occlusion duration was 17.6 min (7.0–25.7). In six dogs that had TCO over 20 min, TCO duration was 20.6, 25.7, 23.8, 25.6, 21.1, and 25.3 min, respectively. No statistically detectable correlation was identified between the degree of CVC compression and TCO duration (Spearman's rank correlation coefficient, ρ = .335, *p* = .161).

Additional procedures were performed in 11 dogs, including en bloc right adrenalectomy (*n* = 5), caudal median sternotomy (*n* = 3), partial pancreatectomy (*n* = 2), lymphadenectomy (*n* = 1), left lateral hepatic lobectomy (*n* = 1), cholecystectomy (*n* = 1), gastrotomy (*n* = 1), and gastropexy (*n* = 1). After mass removal, primary suture repair of the CVC was required in six dogs (31.6%); hemostasis from the CVC was achieved after the removal of both tourniquets in all dogs. In particular, persistent bleeding from the site where the cranial tourniquet was removed was not observed in any case. No intraoperative deaths occurred and all dogs recovered uneventfully from anesthesia. Median operative time among all 19 dogs was 82 min (range, 69–127). Perioperative blood products were administered in 18 of 19 dogs; total perioperative volume was 100 mL (0–400).

### 3.6 Histopathologic findings

HCC was diagnosed in all dogs; the HCCs were classified histopathologically as well differentiated (*n* = 10) or poorly differentiated (*n* = 9). Table 1 summarizes the histologic margin status of all resected masses. Margins were classified as R0 in 14 dogs (73.7%) and R1 in five dogs (26.3%). Median mass weight was 250 g (range, 79–665), and the mass‐to‐bodyweight ratio was 4.5% (range, 1.6–9.9).

Among the 11 dogs that underwent additional procedures, adrenal histopathology in five dogs revealed adrenocortical adenoma in two dogs, adrenocortical carcinoma in one dog, and no lesions in two dogs. Histopathologic examination of the resected pancreas and duodenal lymph node in one dog revealed a stage II insulinoma. In one dog that underwent cholecystectomy, the gallbladder was diagnosed with a mucocele. In one dog that underwent an additional left lateral hepatic lobectomy, the resected lobe contained HCC, consistent with intrahepatic metastasis.

### 3.7 Postoperative progress

Table 1 summarizes the postoperative outcomes. One dog with adrenocortical carcinoma and preoperative hyperadrenocorticism underwent en bloc right adrenalectomy with right divisional hepatectomy and died on postoperative day 2 from an Addisonian crisis. No postoperative hemorrhagic complications were observed. The remaining 18 dogs were discharged, yielding an in‐hospital (short‐term) mortality rate of 5.3%.

Among the 18 discharged dogs, postoperative hospitalization was 9.5 days (range, 6–17), and the duration of follow up at the authors’ hospital before transfer to the referring veterinarian was 24.5 days (range, 15–461). Postoperative complications during hospitalization occurred in 6 of the 18 dogs (33.3%). Persistent postoperative azotemia developed in four dogs and did not develop in 14 dogs. Using the Mann–Whitney *U*‐test, dogs with persistent postoperative azotemia were older than those without postoperative azotemia (14.1 years [12.1–15.6] versus 11.9 years [4.9–14.0]; *p* = .026). No statistically detectable differences were identified between dogs with and without postoperative azotemia in bodyweight (6.4 kg [4.8–13.2] versus 6.3 kg [2.3–12.9]; *p* = .595), TCO duration (15.1 min [7.0–25.6] versus 17.0 min [10.0–25.7]; *p* = .523), or operative time (84 min [69–126] versus 81 min [72–127]; *p* = .915). In contrast, azotemia presented preoperatively in five dogs and resolved after surgery. None of the four dogs that survived concurrent right adrenalectomy developed postoperative hypoadrenocorticism. One dog with hypoglycemia became normoglycemic after hepatectomy, and another dog with concurrent insulinoma remained normoglycemic postoperatively. Delayed incisional healing was noted in one dog that underwent additional caudal median sternotomy but sutures were removed during hospitalization. One dog experienced a transient postoperative seizure of undetermined etiology.

Long‐term follow‐up information was obtained in all 18 discharged dogs. Two dogs returned to the hospital for re‐evaluation and underwent follow‐up CT, which identified lesions that were suspicious for intrahepatic metastasis. In one dog, a lesion in the quadrate lobe suspicious for intrahepatic metastasis was identified 251 days after surgery, and the dog died 562 days after surgery. In another dog, a lesion in the papillary process suspicious for intrahepatic metastasis was identified 426 days after surgery. The lesion was resected 448 days after the initial surgery and histopathology confirmed HCC. The dog died 1151 days after the initial hepatectomy.

At the time of last follow up, 11 dogs had died (median postoperative survival, 729 days [204–1154]). In addition to the two dogs with suspected intrahepatic metastasis, one dog with concurrent insulinoma died 732 days after surgery after refractory hypoglycemia. Other causes of death included lymphoma (*n* = 1), pulmonary adenocarcinoma (*n* = 1), cardiac disease with thrombosis (*n* = 1), refractory protein‐losing enteropathy (*n* = 1), multiple organ failure (*n* = 1), renal failure (*n* = 1), and unknown cause (*n* = 2). Seven dogs were alive without reported recurrence or intrahepatic metastasis at last follow up 95–1355 days after surgery (Table 1). The oldest dog (15.6 years at surgery) remained clinically well 697 days after surgery.

## 4 DISCUSSION

Right‐sided hepatic resections are technically demanding and have historically been associated with higher rates of intraoperative hemorrhage and mortality, although long‐term survival after complete resection of massive HCC can be excellent.^5^, ^6^, ^12^ The primary rationale for applying TCO during right‐divisional hepatectomy is outflow control. The main and accessory right hepatic veins join the CVC within the right divisional parenchyma and must be isolated and divided during resection. Hemorrhage from these hepatic veins and CVC is not controlled by inflow occlusion (e.g., the Pringle maneuver). Previous studies have demonstrated that CVC injury during dissection of right‐sided tumors is a major cause of catastrophic hemorrhage and intraoperative mortality.^5^, ^6^ In this study's cohort, routine placement of cranial and caudal tourniquets to achieve TCO provided a relatively bloodless surgical field, enabling safe identification, ligation, and division of the right and accessory right hepatic veins and facilitating suture repair of small caval defects when present.

Stapling devices are commonly used for hepatic lobectomy. However, in dogs with large right divisional HCC compressing or involving the CVC, there may be insufficient space to safely apply thoracoabdominal or gastrointestinal anastomosis staplers. Forceful stapler application may increase the risk of incomplete resection, stapling through tumor tissue, or iatrogenic CVC injury.^13^ In such cases, careful dissection with individual ligation and division of the hepatic veins is required, and TCO can improve visualization and facilitate safe vascular control and caval repair.

In the present study, R0 resection was achieved in 14 dogs (73.7%), while R1 resection was achieved in five dogs (26.3%). A previous multi‐institutional study reported that complete margins were achieved in 63.9% of dogs with HCC, and the overall recurrence risk was 17.6%.^7^ Overall survival did not differ between complete and incomplete margin groups. Direct comparison is limited by differences in case selection and follow up, these findings support the possibility that, in canine massive HCC, factors other than microscopic margin status may contribute substantially to long‐term outcome.

Median TCO duration in this study (approximately 18 min) fell within the physiologic tolerance reported for short periods of complete CVC occlusion in dogs. An experimental study demonstrated that dogs were able to withstand 20 min of complete abdominal caval occlusion, with hemodynamic recovery after release and collateral channels (notably via the azygos system) contributing to maintenance of circulation.^9^ Although six dogs had TCO durations greater than 20 min, no intraoperative deaths occurred, and no postoperative complications were observed that suggested clinically important intolerance to the TCO intervals used. Nevertheless, the maximum tolerable duration of complete caval occlusion without postoperative morbidity remains unknown, and further investigation is warranted. As TCO transiently reduces venous return, close hemodynamic monitoring and proactive cardiovascular support may be required, and combined fluid and cardiovascular support has been associated with increased odds of short‐term postoperative complications in a contemporary right‐divisional lobectomy cohort.^7^

An alternative strategy may be considered when robust caval‐to‐azygos collateral circulation exists. When preoperative CT confirms extensive collateral pathways from the CVC to the azygos vein, en bloc resection of right‐sided HCC with an involved CVC segment may be feasible without dissection of the right hepatic veins. Experimental work has shown that gradual CVC occlusion can induce caval‐to‐azygos collateral formation,^14^ supporting the physiologic concept of the azygos factor.^8^ Case reports have also described successful en bloc resection of HCC involving the CVC^15^ and resection of an adrenal pheochromocytoma with caval involvement.^16^ In the present study, caval‐to‐azygos collateral circulation was not detected by CT in any dog; therefore, en bloc CVC resection was not planned, and venous back bleeding during dissection was anticipated. Under these circumstances, TCO provided effective outflow control and enabled safe venous dissection and repair. When collateral circulation is present, en bloc resection with the CVC may reduce blood loss and shorten operative time compared with venous dissection under TCO. Accordingly, preoperative CT is crucial for surgical planning.

The proportion of dogs receiving blood products in the present study was higher than that previously reported (21.4%).^7^ This finding should be interpreted cautiously because transfusion thresholds and institutional blood management strategies are not standardized across retrospective studies, and the study cohort represented a particularly high‐risk population with CVC compression or involvement. Transfusion frequency in this series should not be interpreted as a direct surrogate for hemorrhage severity, and direct comparisons of transfusion rates across studies may therefore be misleading.

The perioperative outcomes of this study are consistent with recent reports suggesting that divisional location alone is not necessarily associated with higher postoperative mortality when contemporary techniques and exposure are used, although right divisional resections may still carry higher odds of intraoperative hemorrhage or major vessel injury.^6^, ^8^ In this cohort, TCO appeared to mitigate uncontrolled hemorrhage despite frequent concomitant procedures and a relatively high rate of CVC repair, with hemostasis achieved in all affected dogs.

Although prolonged CVC occlusion could theoretically impair venous return from the kidneys, no statistically detectable difference in TCO duration was identified between dogs with and without persistent postoperative azotemia in this cohort. In the exploratory analysis, dogs with persistent postoperative azotemia were older than those without postoperative azotemia, whereas no statistically detectable differences were identified in bodyweight, TCO duration, or operative time. This finding suggests that older dogs may have reduced renal reserve or greater susceptibility to perioperative renal dysfunction, although causation cannot be inferred from this small retrospective case series. Careful postoperative monitoring of renal function, urine output, hydration status, and systemic blood pressure may be particularly important in older dogs undergoing TCO‐assisted right divisional hepatectomy.

Objective serum chemical reassessment of liver function after discharge was not uniformly available; however, no clinically apparent postoperative hepatic insufficiency was reported by referring veterinarians, and many dogs survived long term. In small animals with HCC, the non‐neoplastic liver parenchyma is typically histologically normal, in contrast with humans in whom cirrhosis frequently precedes HCC.^2^ The long‐term postoperative survival observed in this cohort, together with this histologic feature, may reflect preserved hepatic function of the non‐neoplastic parenchyma in dogs with massive HCC, which often presents as a solitary lesion without diffuse hepatic disease.

Major limitations of this study include its small sample size, retrospective design, and single‐institution setting. Estimated blood loss was not consistently recorded, limiting objective assessment of the hemostatic effect of TCO. Transfusion triggers and thresholds were not standardized and evolved over the study period, limiting interpretation and comparison of transfusion frequency across studies. Most dogs were followed up after discharge at their referring hospitals; therefore, long‐term follow up was heterogeneous and not standardized. Follow‐up imaging and serum chemistry reassessment of liver function were not available for all dogs, and recurrence or metastasis may have been underdetected in some cases. Causes of death were not consistently confirmed (e.g., by necropsy). Prospective multicenter studies with standardized postoperative follow up, including serial liver function testing and imaging, are needed to better define recurrence rates and long‐term outcomes after TCO‐assisted right divisional hepatectomy.

This cohort consisted predominantly of small‐breed dogs, with a median bodyweight of 6.4 kg and a maximum bodyweight of 13.2 kg; the feasibility and complication profile of TCO‐assisted right divisional hepatectomy in larger dogs therefore remain uncertain. No procedure‐specific technical difficulties or complications unique to the heavier dogs in this cohort were identified from the medical records. However, larger body size may influence surgical exposure, operative field depth, vascular caliber, and instrument selection. Further studies including larger dogs are needed to determine whether body size affects the safety or reproducibility of this technique.

In conclusion, TCO‐assisted right divisional hepatectomy provided venous control in selected dogs with massive right‐sided HCC and CVC compression/involvement, with no intraoperative mortality, one in‐hospital death, and postoperative complications in six of 18 discharged dogs. Larger comparative studies with standardized follow up are needed to define perioperative risk, renal outcomes, and long‐term tumor control after this procedure.

## FUNDING INFORMATION

No third‐party funding or support was received for this study or for the preparation or publication of this manuscript.

## CONFLICT OF INTEREST

The authors declare no conflicts of interest.

## INFORMED CONSENT STATEMENT

Informed consent was obtained from all owners prior to the performance of any procedures described in this study.

## Supporting information


**Table S1.** Preoperative blood test of all dogs.

## Data Availability

The data that support the findings of this study are available from the corresponding author upon reasonable request.
