# Supplementary material for: Temporary caval occlusion technique during right divisional hepatectomy for massive hepatocellular carcinoma with caudal vena cava compression/involvement in dogs
Source: Vet Surg. 2026 Jul 8;55(8):1375–86. doi: 10.1111/vsu.70133 (PMC13736194; doi:10.1111/vsu.70133)
Supplement: Supplementary file 1 [file VSU-55-1375-s001.docx]

| Table S1. Preoperative blood test of all dogs | | | | | | | |
| --- | --- | --- | --- | --- | --- | --- | --- |
| Variables | Unit |  | Median |  | Range |  | Reference |
| PCV | % |  | 38 |  | 27 - 53 |  | 37 - 55 |
| Hb | g/dL |  | 12.9 |  | 9.2 - 17.9 |  | 12.0 - 18.0 |
| RBC | 10^6^/μL |  | 5.96 |  | 3.88 - 8.71 |  | 5.50 - 8.50 |
| WBC | /μL |  | 9,400 |  | 4,800 - 22,400 |  | 6,000 - 17,000 |
| Plt | 10^3^/μL |  | 546 |  | 341 - 1,280 |  | 200 - 500 |
| TP | g/dL |  | 7.4 |  | 6.0 - 8.4 |  | 5.0 - 8.0 |
| Alb | g/dL |  | 4.0 |  | 2.6 - 4.6 |  | 2.6 - 4.0 |
| AST | U/L |  | 74 |  | 18 - 291 |  | 17 - 44 |
| ALT | U/L |  | 455 |  | 28 - 2,212 |  | 17 - 78 |
| ALP | U/L |  | 398 |  | 85 - 7,751 |  | 0 - 89 |
| GGT | U/L |  | 10 |  | 2 - 140 |  | 0 - 14 |
| TCho | mg/dL |  | 233 |  | 104 - 445 |  | 115 - 337 |
| TBil | mg/dL |  | 0.1 |  | 0.0 - 0.4 |  | 0 - 0.5 |
| BUN | mg/dL |  | 18.0 |  | 8.4 - 76.8 |  | 9.2 - 29.2 |
| Cr | mg/dL |  | 0.58 |  | 0.35 - 1.22 |  | 0.40 - 1.40 |
| Glu | mg/dL |  | 105 |  | 70 - 123 |  | 75 - 128 |
| Na | mmol/L |  | 147 |  | 143 - 152 |  | 141 - 152 |
| K | mmol/L |  | 4.3 |  | 3.0 - 5.5 |  | 3.8 - 5.0 |
| Cl | mmol/L |  | 109 |  | 99 - 115 |  | 102 - 117 |
| CRP | mg/dL |  | 1.25 |  | 0.05 - 16.00 |  | 0 - 1.00 |
| APTT | sec |  | 14.0 |  | 12.3 - 17.9 |  | 11.0 - 16.0 |
| PT | sec |  | 5.0 |  | 4.2 - 7.1 |  | 5.0 - 6.0 |
| Fib | mg/dL |  | 378.2 |  | 222.3 - 800.0 |  | 128.0 - 420.0 |
| AT | % |  | 189 |  | 116 - 288 |  | 114 - 179 |
| D-dimer | mg/dL |  | 2.82 |  | 0.47 - 24.35 |  | 0 - 2.00 |
| Alb, albumin; ALP, alkaline phosphatase; ALT, alanine aminotransferase; APTT, activated partial thromboplastin time; AST, aspartate aminotransferase; AT, anti-thrombin activity; BUN, blood urea nitrogen; Cr, creatinine; CRP, C-reactive protein; Fib, fibrinogen; GGT, gamma-glutamyl transferase; Glu, glucose; Hb, hemoglobin; PCV, packed cell volume; Plt, platelet count; PT, prothrombin time; RBC, red blood cell count; TBil, total bilirubin; TCho, total cholesterol; TP, total protein; WBC, white blood cell count. | | | | | | | |
